# Heterojunction Devices Fabricated from Sprayed *n*-Type Ga_2_O_3_, Combined with Sputtered *p*-Type NiO and Cu_2_O

**DOI:** 10.3390/nano14030300

**Published:** 2024-02-01

**Authors:** Theodoros Dimopoulos, Rachmat Adhi Wibowo, Stefan Edinger, Maximilian Wolf, Thomas Fix

**Affiliations:** 1 Energy Conversion and Hydrogen Technologies, Center for Energy, AIT Austrian Institute of Technology, Giefinggasse 2, 1210 Vienna, Austria; rachmat.wibowo@ait.ac.at (R.A.W.); stefan.edinger@ait.ac.at (S.E.); maximilian.wolf@ait.ac.at (M.W.); 2 ICube Laboratory, Université de Strasbourg and Centre National de la Recherche Scientifique (CNRS), 23 Rue Du Loess, BP 20 CR, F-67037 Cedex 2 Strasbourg, France; tfix@unistra.fr

**Keywords:** cuprous oxide, gallium oxide, nickel oxide, heterojunctions, sputtering, spray pyrolysis, electron transport layers, hole transport layers, solar cells

## Abstract

This work reports on the properties of heterojunctions consisting of *n*-type Ga_2_O_3_ layers, deposited using ultrasonic spray pyrolysis at high temperature from water-based solution, combined with *p*-type NiO and Cu_2_O counterparts, deposited by radio frequency and reactive, direct-current magnetron sputtering, respectively. After a comprehensive investigation of the properties of the single layers, the fabricated junctions on indium tin oxide (ITO)-coated glass showed high rectification, with an open circuit voltage of 940 mV for Ga_2_O_3_/Cu_2_O and 220 mV for Ga_2_O_3_/NiO under simulated solar illumination. This demonstrates in praxis the favorable band alignment between the sprayed Ga_2_O_3_ and Cu_2_O, with small conduction band offset, and the large offsets anticipated for both energy bands in the case of Ga_2_O_3_/NiO. Large differences in the ideality factors between the two types of heterojunctions were observed, suggestive of distinctive properties of the heterointerface. Further, it is shown that the interface between the high-temperature-deposited Ga_2_O_3_ and the ITO contact does not impede electron transport, opening new possibilities for the design of solar cell and optoelectronic device architectures.

## 1. Introduction

By virtue of their versatile, wide-ranging electronic properties, metal oxide semiconductors are indispensable materials for a wide range of devices, including photovoltaic (PV) and photoelectrochemical cells, organic light-emitting diodes (OLEDs) and photodetectors, or power electronics components. They provide functionalities as transparent electrodes, charge-carrier-selective transport or injection layers, as well as light absorbers, depending on their electrical conductivity, work function, and bandgap. Nickel oxide (NiO), a *p*-type wide bandgap (3.4–3.7 eV) semiconductor, bears a high potential to replace organic hole transport layers in perovskite and organic photovoltaic devices, as elaborated in many recent reviews [1,2,3,4,5]. Cuprous oxide (Cu_2_O), also a *p*-type material, with a bandgap of 1.9–2.6 eV, has been introduced as a hole-transport layer in perovskite cells [6], recently achieving very promising efficiency and stability results [7]. Because of its relatively low bandgap, it has been widely investigated as a light absorber in all-oxide solar cells [8], as well as in photoelectrochemical cells for water splitting [9]. Gallium oxide (Ga_2_O_3_), on the other hand, is an *n*-type, wide-bandgap (4.8–5.0 eV) semiconductor that has demonstrated high potential in solar cells when combined with Cu_2_O, as will be elaborated later. However, it has also found implementation as an electron-transport layer in perovskite solar cells [10]. Heterojunctions between Ga_2_O_3_ and NiO or Cu_2_O are of interest, both from a fundamental and applications points of view.

Ga_2_O_3_/NiO junctions have been investigated in the literature, mainly for applications in power electronics, as summarized in a recent review [11]. In these devices, NiO thin films have been deposited on single-crystal Ga_2_O_3_ substrates or thick (several micrometers) Ga_2_O_3_ layers [12,13,14,15], with different techniques, including sol-gel [12], atomic layer deposition (ALD) [13], and radio-frequency (RF) sputtering [13,14]. The reverse architecture has also been reported: Wang et al. [16] sputter-deposited 4 µm thick films of NiO, combined with sputtered 200 nm thick Ga_2_O_3_ films, for use as self-powered photodetectors. In all of these works, a type II band alignment at the heterojunction has been shown, with conduction band offset (CBO) between 0.9 and 2.7 eV and valence band offset (VBO) between 2.1 and 3.6 eV [12,13,14,15,16,17]. This wide spread of values shows the sensitivity of the energy band alignment on the material’s processing, crystal orientation, and other factors. However, such thick films cannot be applied as electron and hole transport layers in PV or OLED devices and reducing the thickness to the tens of nanometers regime—or further—is challenging for the structural integrity of the layers and, therefore, the operation of the device. 

The interest in Cu_2_O/Ga_2_O_3_ heterojunctions has increased in the last decade, since Minami et al. reported high-efficiency (~5.4%) Cu_2_O/Ga_2_O_3_ solar cells, using thermal oxidation of Cu sheets at high temperatures for the preparation of Cu_2_O and pulsed laser deposition for Ga_2_O_3_. The high efficiency is caused by the optimum conduction band alignment between the *p* and *n* layers [18]. A bit later, Lee at al. reported 1.2 V open circuit voltage and efficiency of ~4% in Cu_2_O/Ga_2_O_3_ heterojunctions with electrodeposited Cu_2_O and Ga_2_O_3_ fabricated by ALD, with optimized band alignment and passivation of interface defects [19]. Chua et al. showed that, when growing Ga_2_O_3_ by ALD on top of Cu_2_O grown by chemical vapor deposition (CVD), exposure to air is of vital importance for the transport properties, as a CuO layer forms on the Cu_2_O. By avoiding air exposure between the deposition of the two layers, they could enhance the open circuit voltage from 1.4 V to ~1.8 V [20]. From these works, one can conclude that, from the point of view of interface stoichiometry control, it is advantageous to deposit Cu_2_O on Ga_2_O_3_ and not vice versa, unless the surface oxidation of Cu_2_O can be avoided. Benz et al. fabricated both oxide layers by sputtering and investigated the VBO and CBO at the interface. They found that *α*-Ga_2_O_3_/Cu_2_O has the lowest CBO of ~0.2 eV and a VBO of ~3.2 eV, while, in *β*-Ga_2_O_3_/Cu_2_O junctions, the CBO increases to ~1.3 eV and the VBO to ~3.7 eV. To this respect, they claimed that the use of *α*-Ga_2_O_3_ bears the highest potential for application in solar cells in combination with Cu_2_O [21]. Their work, however, did not show electrical characterization of the junctions. 

The present work reports for the first time the properties of heterojunctions composed of *n*-type Ga_2_O_3_ layers deposited by ultrasonic spray pyrolysis [22], in combination with RF- and DC-sputtered *p*-type NiO and Cu_2_O counterparts, respectively. The structural, optical, and electronic properties of the single layers are investigated before the analysis of the electrical transport characteristics of the junctions in the dark and under simulated solar light, yielding information about the energy band alignment in the two junction types. 

## 2. Materials and Methods

Three types of substrates were used in this study: the first was uncoated borosilicate glass (NEXTERION^®^ D, 1.0 mm thickness, cut in 25 × 25 mm size, Schott, Jena, Germany), used for the deposition of Ga_2_O_3_ by spray-pyrolysis, due to its superior thermal stability. The second was uncoated, soda-lime, microscope slides (size: 25 × 25 × 1.0 mm, Menzel Gläser, Braunschweig, Germany), used for the deposition of the sputtered layers, and the third was ITO-coated glass (size: 25 × 25 × 1.1 mm, Product No. 703192, Merck, Darmstadt, Germany) with a sheet resistance of 8–12 Ω/sq, used for the deposition of devices. 

For the patterning of the ITO-coated substrates, the following procedure was followed: polyimide film tape (Kapton^®^, DuPont Teijin Films, Chester, VA, USA) with a width of 12.5 mm was used to cover a middle stripe of the ITO, before immersing the substrate into a 9 M HCl aqueous solution (1:1 dilution), etching away the ITO from the uncovered area. After the etching, the substrate was rinsed with ultrapure water (resistivity: 18 MΩ·cm, Arium^®^, Göttingen, Germany) and the tape was removed. The substrate was then thoroughly cleaned by ultrasonication in a bath with ultrapure water and cleaning concentrate (Helmanex^TM^ III, Merck, Darmstadt, Germany), under sonication at 50 °C, followed by sonication in ultrapure water and, finally in isopropanol (each step for a 15 min duration), before being blown-dry in nitrogen stream. The same cleaning procedure was applied for the bare glass substrates.

The substrates were then transferred to the spray pyrolysis setup (Sono-Tek ExactaCoat^®^, equipped with a Sono-Tek Impact^®^ ultrasonic nozzle operating at 120 kHz, Sono-Tek Corporation, Milton, NY, USA), where they were heated to 380 °C and coated with Ga_2_O_3_, following the process described in [22]. The layer thickness range was between 12 and 30 nm. For the Ga_2_O_3_ deposition on the ITO substrate, a layer thickness of 15 nm was chosen. A 3 mm wide stripe was left uncoated on one side of the ITO substrate to be later used as back-side contact. After the Ga_2_O_3_ deposition, the substrates were left to cool down to ~50 °C, before being taken off the hotplate. After the spray-pyrolysis, due to the applied thermal budget, the ITO substrate’s sheet resistance increased to 51 ± 6 Ω/sq. 

For the sputtering, a Leybold Univex cluster tool was used (Leybold, Cologne, Germany). All layers were deposited without substrate heating at a substrate-to-target distance of ~9 cm. The deposition of NiO was carried out using a NiO target (101.6 mm diameter, 99.99% purity, AJA Int., Scituate, MA, USA), mounted on an RF magnetron source. The applied sputter power was 200 W and pure Ar was used as process gas at a pressure of 10 µbar, resulting in a sputter rate of 0.11 nm/s. Layers of 20–40 nm in thickness were deposited. The deposition of Cu_2_O was carried out using a metallic Cu target (101.6 mm diameter, 99.995% purity, Materion, Mayfield Heights, OH, USA), mounted on a DC magnetron source. A sputter power of 80 W was applied for the deposition in an atmosphere of Ar/O_2_:80/20 at a pressure of 10 µbar. Prior to each deposition process, the target was sputtered in pure Ar atmosphere at 120 W and 5 µbar pressure for at least 10 min, followed by 5 min in Ar/O_2_ atmosphere at 80 W. This step served to condition and clean the target from the oxide and achieve reproductible deposition results with pure Cu_2_O phase. At these conditions, the sputter rate was 0.74 nm/s. The deposition of the device contacts was carried out using an Au target (76 mm diameter, 99.99% purity, Neyco, Vanves, France) at 20 W and 2 µbar Ar pressure, resulting in a rate of 0.8 nm/s. The contacts were sputtered through a shadow mask, defining active device areas of 5.73 mm^2^ and 10.43 mm^2^. 

The sputter deposition rates for NiO and Cu_2_O were extracted by measuring the layer thickness (step height) using a surface profilometer (Alpha-Step^®^ IQ, KLA-Tencor, Milpitas, CA, USA). The morphology of the layers was measured by scanning electron microscopy (SEM) (Zeiss Ultra 40, Zeiss, Oberkochen, Germany) with a beam accelerating voltage of 5 kV and an in-lens detector. The surface topography was evaluated by atomic force microscopy (AFM) (PicoPlus, Molecular Imaging, Tempe, AZ, USA) in tapping mode, using SSS-NCHR probes (Nanosensors™, Neuchatel, Switzerland). The open-source software Gwyddion, version 2.61, was used to plot and analyze the AFM data. Structural characterization of the layers was realized using a grazing incidence X-ray diffractometer (XRD) (ARL Equinox 100, Thermo Fisher Scientific, Waltham, MA, USA) at an angle of 5 °, with Cu-*K*_α_ radiation. The analysis of the diffractograms was conducted using the Match! Software, version 3.14 (Crystal Impact, Bonn, Germany), with reference databases from Crystallographic Open Database (COD).

Transmission and reflectance spectra were recorded by a Fourier transform spectrometer (FTS) (Bruker, Billerica, MA, USA, Vertex 70) equipped with a halogen lamp as a source of unpolarized light (64642 HLX, Osram Licht, Munich, Germany). Direct transmittance was measured at normal incidence in reference to air and reflectance was measured at an incidence angle of 13° in reference to a calibrated mirror (STAN-SSH-NIST, Ocean Optics, Orlando, FL, USA). A GaP and a Si diode detector were used for the spectral ranges of 303−588 nm and 500−1205 nm, respectively. 

Optical simulations were performed using a transfer matrix method (TMM) algorithm, described by Ebner et al. [23]. The sheet resistance was determined using an in-line four-point probe (Nagy SD–600, Nagy Instruments, Gäufelden, Germany). Current density–voltage (*j-V*) curves of solar cells were obtained with two-point measurements, using a semiconductor parameter analyzer (4156C, Agilent Technologies, Santa Clare, CA, USA) under dark and AM1.5G-simulated illumination (LOT-Oriel solar simulator, Darmstadt, Germany). For the capacitance–voltage (*C-V*) measurements, an LCR Meter was used (4284A, Agilent Technologies, Santa Clare, CA, USA). 

For the determination of the work function (WF) and ionization energy (IE) of the layers, Kelvin probe and ambient pressure photoemission spectroscopy (APS) were performed in air (KP Technology, Wick, UK, APS03). A 2 mm diameter Au-coated tip was calibrated using air photoemission. The WF of the material was determined by measuring the contact potential difference between the Kelvin probe tip and the surface of the sample. The IE was measured using the same system by photoelectron emission. Finally, for the electrochemical impedance spectroscopy (EIS), a 3-electrode cell was used, with the coated sample serving as a working electrode, platinized Ti mesh as a counter electrode, and Ag/AgCl as reference. The cell was connected to a potentiostat (Vionic, Metrohm, Herisau, Switzerland). A 0.5 M Na_2_SO_4_ aqueous electrolyte was used. 

## 3. Results

### 3.1. AFM and SEM Characterization

**Figure 1:**
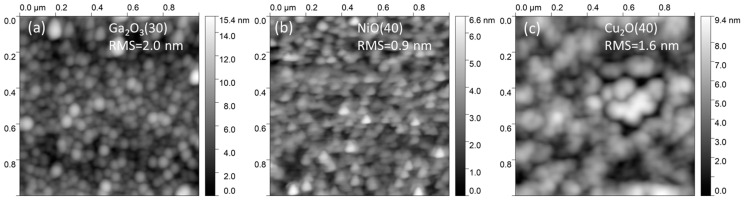
AFM images of single layers of: (**a**) Ga_2_O_3_(30), (**b**) NiO(40), and (**c**) Cu_2_O(40), along with the corresponding RMS roughness values.

AFM characterization was realized for layers deposited on glass substrates. All layers have low roughness, with fine grain structure, making them suitable for implementation in ultra-thin solar cells and optoelectronic devices. The largest roughness is measured for Ga_2_O_3_: the 30 nm thick layer yields RMS roughness of 2.0 nm (Figure 1a), with the background roughness of the borosilicate glass being 0.3 nm (Figure S1a). The lowest roughness is measured for NiO: the 40 nm thick layer yields RMS roughness of 0.9 nm (Figure 1b), the same as the background roughness of the glass substrate (Figure S1b). The RMS value for the 40 nm thick Cu_2_O is 1.6 nm (Figure 1c), with a grain size considerably larger than for Ga_2_O_3_ and NiO.

**Figure 2:**
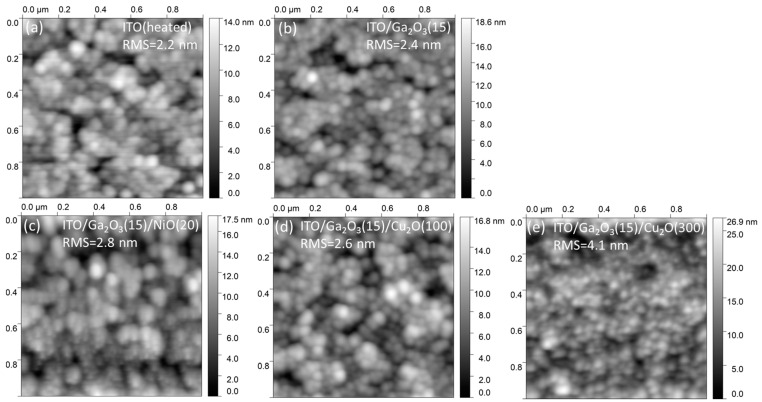
AFM images of the: (**a**) ITO substrate, (**b**) ITO/Ga_2_O_3_(15), (**c**) ITO/Ga_2_O_3_(15)/NiO(20), (**d**) ITO/Ga_2_O_3_(15)/Cu_2_O(100), and (**e**) ITO/Ga_2_O_3_(15)/Cu_2_O(300) stacks, along with the obtained RMS roughness values.

The heterojunction multilayers also feature low roughness. Figure 2 shows the roughness evolution with the sequential deposition of layers. The ITO back electrode (heated to the temperature used for the Ga_2_O_3_ deposition, i.e., 380 °C) has an RMS value of 2.2 nm. The deposition of 15 nm Ga_2_O_3_ atop, leads to a marginal increase in the RMS to 2.4 nm. The RMS further increases moderately with the deposition of the 20 nm NiO, reaching 2.8 nm. On the other hand, 100 nm of Cu_2_O brings the RMS to 2.6 nm, increasing to 4.1 nm when a 300 nm Cu_2_O film is deposited. The AFM images suggest that, with the increase in Cu_2_O thickness, the film acquires a more compact character, which helps to keep the overall roughness of the stack at low levels. 

**Figure 3:**
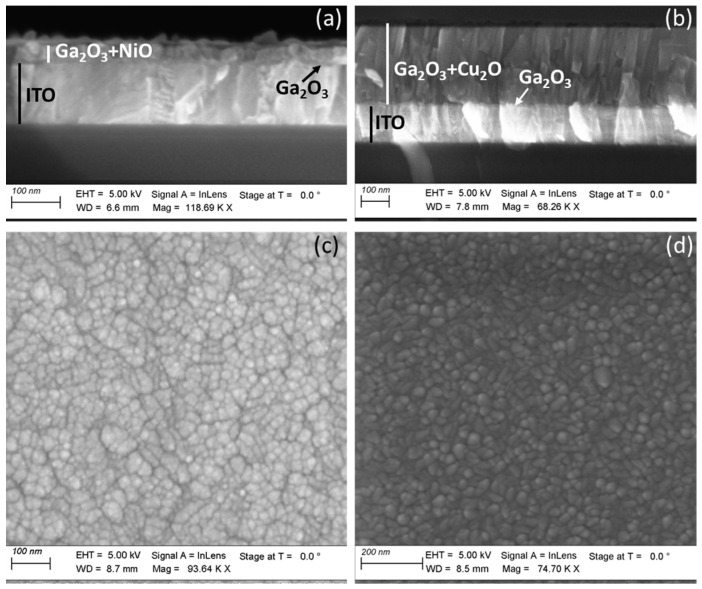
Cross-section and plain-view SEM images of the (**a**), (**c**) ITO/Ga_2_O_3_(15)/NiO(20) and (**b**), (**d**) ITO/Ga_2_O_3_(15)/Cu_2_O(300) stacks.

The compact character of the layers can be further evidenced in the cross-section SEM images, shown in Figure 3. One can clearly distinguish the ITO layer with a thickness of ~125 nm, followed by the compact and continuous Ga_2_O_3_ interfacial layer and the NiO, having a combined thickness of ~35 nm (Figure 3a). On the other hand, for the sample in Figure 3b, the Cu_2_O layer has a thickness of ~300 nm. The plain-view SEM images (Figure 3c,d) show again very different grain structures between the ultra-thin NiO and the thick Cu_2_O.

### 3.2. Structural Characterization

Single NiO and Cu_2_O layers of different thicknesses were deposited on plain glass substrates for XRD characterization. As described in [22], relatively thick (>150 nm) spray-pyrolyzed Ga_2_O_3_ layers adopt the monoclinic *β*-Ga_2_O_3_ structure, with predominant (111) texturing. However, GIXRD diffractograms of thin films of the order used in this work (15–30 nm) show no reflection peaks (apart from the broad background of the glass substrate on which they were deposited). 

**Figure 4:**
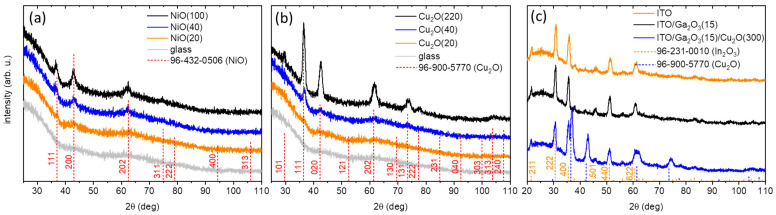
GIXRD patterns of the (**a**) NiO and (**b**) Cu_2_O single films of different thicknesses. (**c**) GIXRD patterns of the ITO, ITO/Ga_2_O_3_(15), and ITO/Ga_2_O_3_(15)/Cu_2_O(300) stacks.

For the NiO layers, down to a thickness of 20 nm, crystalline structure reflections can be observed (Figure 4a). For the 100 nm film, the pattern is composed of the (111), (200), (202), and (311) peaks, with the (200) and (111) being the most prominent, in agreement with the reference for cubic NiO ($Fm\bar{3}m$, COD: 96-432-0506). For the 40 nm film, the (200), (111), and (202) peaks are clearly visible and, even for the 20 nm thick film, the (200) peak can still be distinguished. The structure of the RF-sputtered films agree with various reports from the literature implementing DC or RF mode sputtering in Ar and Ar/O_2_ atmosphere [24,25,26]. 

The Cu_2_O 220 nm thick film yields a pattern that perfectly matches the cuprite cubic reference ($Pn\bar{3}m$, COD: 96-900-5770), with high-intensity (111), (020), and (202) peaks, underlining the polycrystalline character of the deposit (Figure 4b). Similar results were obtained by reactive DC sputtering of Cu_2_O from a Cu target in the literature [27]. A clear pattern is also distinguished for the 40 nm film, with the aforementioned reflections present. For the 20 nm film, no reflections are visible. In conclusion, the GIXRD characterization demonstrates the crystalline nature of the NiO and Cu_2_O, as well as the phase purity, as no foreign reflections were detected. 

Figure 4c shows the GIXRD pattern of the ITO substrate, corresponding to the cubic structure ($Ia\bar{3}$, COD: 96-231-0010). After the deposition of the Ga_2_O_3_ layer at high temperature, the reflection peaks of the ITO remain unchanged and no additional peaks can be observed. For the complete ITO/Ga_2_O_3_/Cu_2_O(300) multilayer, both In_2_O_3_ and Cu_2_O patterns can be clearly distinguished. The same polycrystalline pattern is obtained for the Cu_2_O on the ITO/Ga_2_O_3_ substrate as on the glass, which is not always the case for sputtered Cu_2_O films [28]. It is noted, however, that, in this case, the Cu_2_O peaks are all slightly shifted to the right with respect to the reference and the single films, which can be attributed to an induced stress when the layer is deposited on top of the ITO/Ga_2_O_3_ substrate.

### 3.3. Optical Characterization

**Figure 5:**
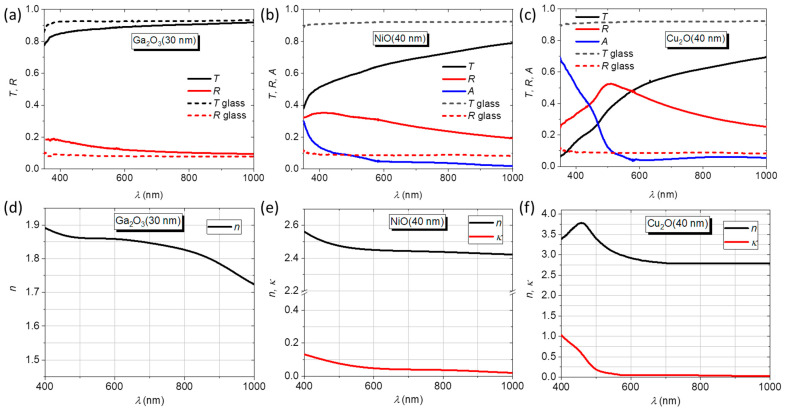
Transmittance (*T*), reflectance (*R*), and absorbance (*A*) spectra for single layers of (**a**) Ga_2_O_3_(30), (**b**) NiO(40), and (**c**) Cu_2_O(40), together with the *T* and *R* of the respective glass substrates. The second row shows the calculated refractive index and extinction coefficient for (**d**) Ga_2_O_3_, (**e**) NiO, and (**f**) Cu_2_O.

To extract the refractive index from optical spectra, single layers of the oxides with a thickness below 50 nm were deposited to avoid light interference patterns in the spectra that can complicate the index determination procedure. Figure 5a–c show transmittance (*T*), reflectance (*R*), and absorbance (*A* = 1 − *T* − *R*) spectra for the Ga_2_O_3_(30), NiO(40), and Cu_2_O (40) layers, respectively (for Ga_2_O_3_, A is practically zero). All spectra are referenced to air. Each graph also includes the *T* and *R* spectra of the glass substrates. The Ga_2_O_3_ layer is highly transparent over the whole spectrum, while NiO presents considerable absorption losses for wavelengths < 600 nm. In the same spectral region, as expected, Cu_2_O presents strong absorption. Considering the refractive index of the glass *n*_G_ = 1.52 and air as the surrounding medium (*n*_air_ = 1.00), the transfer matrix method (TMM) was used to calculate the complex refractive index of the materials in the range 400–1000 nm, as shown in Figure 5d–f. Due to the negligible absorption, only the real part of the refractive index is plotted for Ga_2_O_3_, with *n* decreasing continuously with the wavelength from 1.89 at 400 nm to 1.72 at 1000 nm. The results are in line with the literature reports for Ga_2_O_3_ layers fabricated by different techniques like sputtering [29] or plasma-enhanced atomic layer deposition [30]. 

A significantly larger *n* is calculated for NiO, decreasing from 2.57 at 400 nm to 2.42 at 1000 nm. The extinction coefficient *κ* decreases with the wavelength from *κ* = 0.14 at 400 nm to 0.017. Likewise, the refractive index of the NiO layer agrees with literature results from spectroscopic ellipsometry measurements of reactively sputtered NiO films [31]. For Cu_2_O, *n* maximizes to the value of 3.79 at 450 nm and reaches a plateau at 2.79 above 700 nm. The extinction coefficient has the value of 1.00 at 400 nm, dropping to 0.055 for *λ* > 600 nm. The extracted complex refractive index of Cu_2_O agrees very well with reported values for bulk material measured by spectroscopic ellipsometry [32], indicating the high optical quality of the sputtered films. 

**Figure 6:**
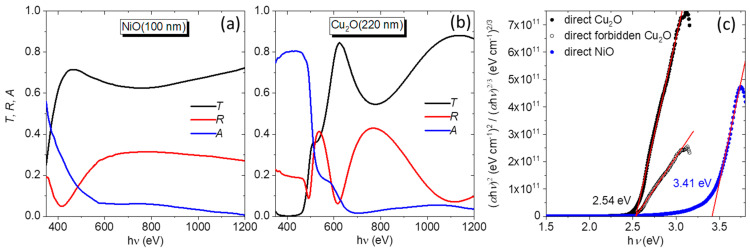
Transmittance, reflectance, and absorbance (*A* = 1 − *T* − *R*) spectra for (**a**) NiO(100) and (**b**) Cu_2_O(220) layers. (**c**) Tauc plots for the bandgap estimation of the NiO and Cu_2_O layers, assuming direct and direct forbidden transition for Cu_2_O and direct transition for NiO. Also shown are the linear fits in the band-edge regions (red lines).

The bandgap of the sprayed Ga_2_O_3_ was previously reported to be ~5.0 eV [22]. Here, the bandgap values of NiO and Cu_2_O are calculated from the optical spectra of thicker films (Figure 6a,b), using the Tauc plot method [33]. For this, the absorption coefficient is obtained from the relation [34]:

$$\alpha =\frac{1}{t}\ln\left(\frac{1-R}{T}\right),$$

where *t* is the film thickness. The relation ${\left(\alpha h\nu \right)}^{m}=C(h\nu -E_{g})$ is used to fit the linear part of the plot ${\left(\alpha h\nu \right)}^{m}$ vs. hν, corresponding to the band-edge of the material. The factor *m* assumes the value of 2 for direct bandgap, 1/2 for indirect bandgap, and 2/3 for direct forbidden transition. 

For NiO (Figure 6c), the best linear fit of the band edge is obtained for *m* = 2 (direct bandgap), giving rise to a bandgap value of 3.41 eV. This value is at the low end of the reported literature range for sputtered NiO films, which is 3.34–3.71 [24,35,36,37]. For Cu_2_O (Figure 6c), an equally satisfactory linear fit in the band-edge region can be obtained if a direct allowed (*m* = 2) or direct forbidden (*m* = 2/3) transition is considered, with the latter being a more realistic assumption for this material [38]. In both cases, the bandgap value is $E_{g}=2$.54 eV, as can be seen in Figure 6c. This value is in the upper end of the range reported for Cu_2_O layers, prepared by sputtering, which is between 2.18 and 2.58 eV [39,40,41]. A general conclusion in the literature is that the widening of the Cu_2_O bandgap relates to the reduction in defects and enhanced crystallization, which were achieved with the help of annealing [40,41] or the use of mixed O_2_-N_2_ reactive gas during deposition [39]. 

**Figure 7:**
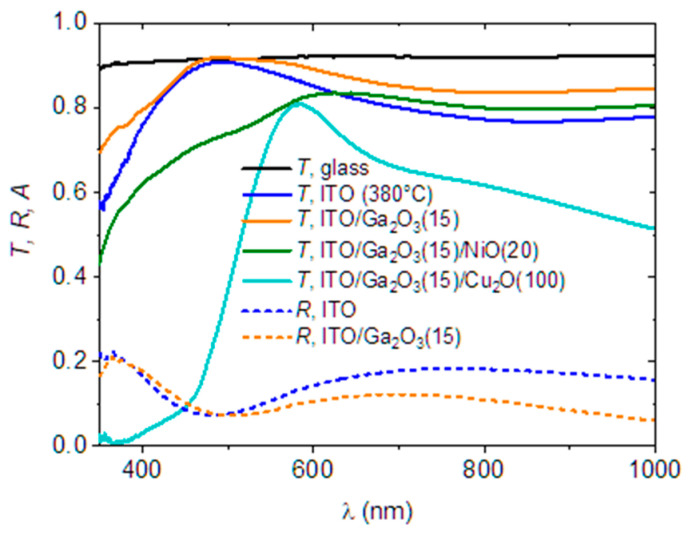
Transmittance, reflectance, and absorbance spectra for the heterojunctions at sequential stages of their deposition.

Figure 7 shows optical spectra for the heterojunctions at sequential stages of their deposition. Interestingly, the transmittance of the glass/ITO substrate that is coated with the Ga_2_O_3_(15) layer is higher than the transmittance of the uncoated ITO (subjected to the thermal stress of the spray deposition). This is due to a reduction in the reflectance losses, as seen from the comparison of the two reflectance spectra in Figure 7. The deposition of the NiO(20) on the Ga_2_O_3_ reduces considerably the transmittance for *λ* < 620 nm due to the absorption of the NiO layer. The transmittance after the deposition of 100 nm Cu_2_O shows enhanced absorption for *λ* < 470 nm and a subsequent gradual increase in the *T* as the band edge of the material is approached. 

### 3.4. Electronic Properties Characterization

The Kelvin probe measurements yielded work function (WF) values of 4.8, 4.9, and 4.2 eV for Cu_2_O, NiO, and Ga_2_O_3_, respectively. The WF for Ga_2_O_3_ is 0.7 eV higher than the one previously extracted from UPS measurements [22]. It is known, however, that the two techniques give rise to distinctive values, as UPS measurements take place in ultra-high vacuum and Kelvin probe in air. Furthermore, the former gives a minimum value of the WF (as it is estimated by comparing the Fermi energy and the low-energy cut-off of the secondary electrons), while the latter gives an average over the probed electrode area. In addition, as pointed out in the introduction, the air-exposed surface of Cu_2_O is covered with an atomically thin layer of CuO, which has a smaller WF than Cu_2_O [42]. For this reason, the extracted value is only representative of the air-exposed Cu_2_O but not of the surface formed upon Cu_2_O deposition on Ga_2_O_3_ under vacuum. The ionization energy (IE) values, extracted from APS measurements, are 5.2 eV for both Cu_2_O and NiO samples. For the Ga_2_O_3_, the IE cannot be extracted from the APS, as it is below the measurable limit.

**Figure 8:**
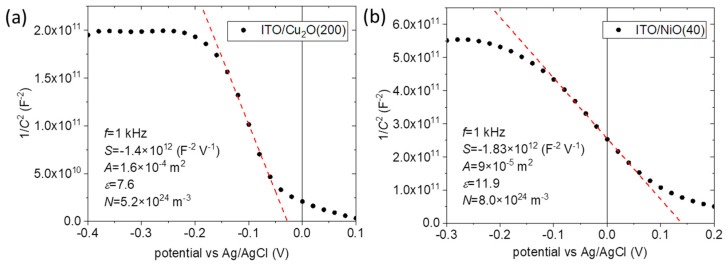
Mott–Schottky plots for the (**a**) ITO/Cu_2_O(200) and (**b**) ITO/NiO(40) samples. The red lines are linear fits.

From the EIS measurements, at a frequency of *f* = 1 kHz, Mott–Schottky plots were constructed (Figure 8), showing negative slopes and, therefore, *p*-type conductivity for both Cu_2_O and NiO layers. The hole carrier density N was extracted from the formula:

$$N=\frac{2}{qA^{2}\varepsilon_{0}\varepsilon \cdot S},$$

where q is the electron charge, A the electrode area in the electrolyte, $\varepsilon_{0}$ the vacuum permittivity, ε the permittivity of the semiconductor (7.6 for Cu_2_O and 11.9 for NiO) [43], and S the slope of the linear fit of the Mott–Schottky plot. Carrier density values of 5.2 × 10^24^ m^−3^ and 8.0 × 10^24^ m^−3^ for Cu_2_O and NiO, respectively, were extracted, as shown in Figure 8a,b, respectively. These high carrier density values are in agreement with other works in the literature on sputtered Cu_2_O [44] and NiO [31] films.

### 3.5. Heterojunction Charaterization

For the heterojunctions, a standard Ga_2_O_3_ thickness of 15 nm was selected, based on an initial screening of the thickness-dependent performance, described in the Supplementary Information (Figure S2). 

**Figure 9:**
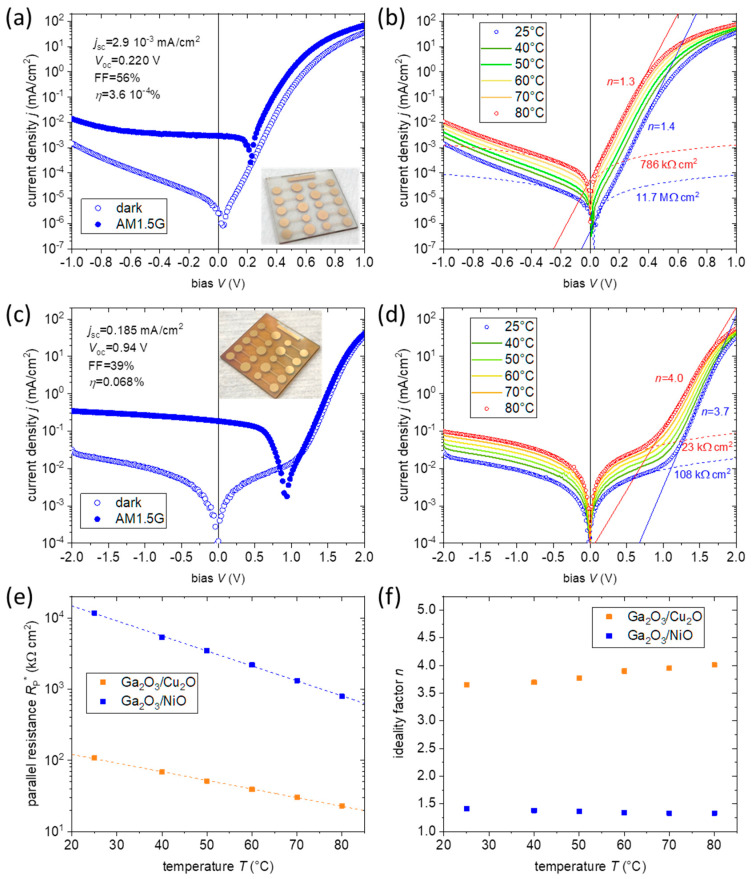
(**a**) *j-V* curves in dark and under illumination for the Ga_2_O_3_(15)/NiO(20) junction together with a photo of a sample. (**b**) Dark *j-V* curves of the Ga_2_O_3_(15)/NiO(20), as a function of the temperature, with the fitting for ideality factor and parallel resistance for *T* = 25 °C and 80 °C. (**c**) *j-V* curves in dark and under illumination for the Ga_2_O_3_(15)/Cu_2_O(300) junction together with a photo of a sample. (**d**) Dark *j-V* curves of the Ga_2_O_3_(15)/Cu_2_O(300), as a function of the temperature, with the fitting for ideality factor and parallel resistance for *T* = 25 °C and 80 °C. (**e**) Plot of the parallel resistance of the devices as a function of the temperature. (**f**) Plot of the ideality factor of the devices as a function of the temperature.

The properties of the ITO/Ga_2_O_3_(15)/NiO(20)/Au(100) heterojunctions were analyzed under dark and illuminated conditions. For a typical device, dark and illuminated *j-V* curves are shown in Figure 9a in a semilogarithmic scale (inset shows photo of a sample). The dark *j-V* shows large rectification (>1000 at |*V*| = 0.5 V), which demonstrates the formation of a high-quality *n/p* junction between the ultra-thin Ga_2_O_3_ and NiO layers. The dark *j-V* shows a low turn-on bias of ~30 mV in the forward direction (positive bias applied on the Au contact). The ideality factor *n* of the junction can be extracted from the fitting of the *j-V* curve for intermediate forward bias, in the range 0.16–0.36 V, using the diode equation:

$$j=j_{0}\cdot \exp\left[\frac{qV}{nkT}\right],$$

which is derived as an approximation for the intermediate bias region of the general equation:

$$j=j_{0}\cdot \left\{\exp\left[\frac{q\left(V-j\cdot R_{S}^{*}\right)}{nkT}\right]-1\right\}+\frac{V-j\cdot R_{S}^{*}}{R_{P}^{*}},$$

where $j_{0}$ is the saturation current density, $R_{S}^{*}=R_{S}\cdot A$ and $R_{P}^{*}=R_{P}\cdot A$, with $R_{S}$ and $R_{P}$ being the series and the parallel resistance, respectively, and A the junction area, *k* the Boltzmann constant, and *T* the temperature. The first term in the above equation corresponds to the exponential diode current, whereas the second term is the shunt current, which can be approximated as $V/R_{P}^{*}$ for reverse and low forward bias. The average ideality factor and standard deviation over five devices is *n* = 1.6 ± 0.2. The ideality factor is, therefore, as expected from the Sah–Noyce–Shockley theory [45], in the regime between 1 and 2. The Ga_2_O_3_/NiO junction is a wide-gap, type II heterojunction with a large CB offset and an even larger VB offset. These large offsets block currents, so interface recombination is regarded as the dominant carrier transport channel across the heterojunction. The temperature dependence of the dark *j-V* characteristics was measured at 25, 40, 50, 60, 70, and 80 °C and the results are shown in Figure 9b. From the fitting in the intermediate forward voltage range, it is obtained that the ideality factor only slightly decreases with increasing temperature, as expected for the generation-recombination type of carrier transport. For the device shown in Figure 9, n decreases from 1.4 at ambient temperature to 1.3 at 80 °C (Figure 9b,f). From the fitting of the dark *j-V* curves in the region (−0.2, 0.1) V, a high parallel resistance is extracted, with a value of 11.7 MΩ cm^2^ (Figure 9b). For all measured NiO solar cells, the parallel resistance was in the MΩ cm^2^ range at ambient temperature. The illuminated curves show a very low short-circuit current density of *j*_sc_ = (3.0 ± 0.5) µA/cm^2^, as expected from the minimal absorption of the high bandgap *n* and *p* counterparts. The open circuit voltage, *V*_oc_, is (224 ± 11) mV. 

The *j-V* characteristics of the Cu_2_O-based heterojunctions were also analyzed in a similar manner. In Figure 9c,d, typical curves for the ITO/Ga_2_O_3_(15)/Cu_2_O(300)/Au(100) stack under dark and illuminated conditions are plotted in a semilogarithmic scale (inset shows photo of a sample). The dark *j-V* shows large rectification at much higher bias than for the NiO junctions (>1000 at |*V*| = 2 V). A low parallel resistance dominates the junction properties at low and intermediate bias regimes. From the fitting of the *j-V* curves in the regime (−0.5, 0.5) V, a parallel resistance of 108 kΩ cm^2^ is extracted at ambient temperature. For all solar cells of this type, the *R*_P_ was in the range 60–170 kΩ cm^2^. 

Parallel current can be caused by the trapping and de-trapping of the carriers at defect states in the space charge region of the device. These defects can act either as recombination centers or traps depending upon the relative capture sections of the electrons and holes [46]. When charges entering the space charge region are captured in these states, they can further jump from one state to the other through tunneling or being thermally re-emitted into the conduction or valence band or to another such state. These mechanisms contribute to the *R*_P_, while the recombination mechanism contributes to the exponential term in the current. The thermal (re)emission from the traps depends on the temperature as, with increasing temperature, the rate of trap depopulation is increased, giving rise to more free carriers. This process is described by the equation:

$$dN_{t}\left(T\right)={-N}_{t}\left(T\right)\nu \exp\left(-\frac{E}{kT}\right)dt,$$

where $N_{t}\left(T\right)$ is the number of trapped carriers at temperature T, E the energy of the state, and ν the attempt-to-escape frequency, which is proportional to the density of states of the conduction or valence band, the capture cross-section of electrons or holes, and their thermal velocity. As elaborated in [46], this process leads to an ohmic behavior of the shunt current with respect to the bias voltage and an exponential dependence with respect to the temperature. This agrees with the observed exponential dependence of the parallel resistance on the temperature for both types of solar cells, as shown in Figure 9e. 

Another important characteristic of the Cu_2_O heterojunction *j-V* curves is the high ideality factors (>3.5) obtained in the exponential current growth regime. In this case, the ideality factor shows a moderate increase from 3.7 at ambient temperature to 4.0 at 80 °C, as can be seen from Figure 9f. Such high ideality factors were reported for inorganic [47,48,49], organic [50], and perovskite devices [51] and have been attributed to different origins, such as (a) the existence of other rectifying junctions in the stack, (b) shunts and defects, especially at the borders of the junction areas, (c) transport across tunnel barrier, or (d) energy state disorder. Rectifying junctions can be, indeed, formed at either contact: ITO/Ga_2_O_3_ or Cu_2_O/Au. A rectifying ITO/Ga_2_O_3_ junction cannot be responsible for the large ideality factor, as this would also influence the ideality factor of the heterojunctions employing NiO, which, as shown before, is not the case. A Schottky junction between Cu_2_O and Au is also not probable in view of the favorable energy band alignment. However, to exclude this possibility, heterojunctions with a NiO(20) layer inserted between the Cu_2_O and Au were deposited. The NiO/Au contact should be ohmic, as the Ga_2_O_3_/NiO junctions have low ideality factors. However, the junctions with the inserted NiO have also shown ideality factors in the same range as the ones without NiO (Figure S3). From these experiments it can be concluded that contact-related rectifying junctions cannot be at the origin of the high ideality factors. Edge shunts related to the device fabrication can contribute to increasing the ideality factor; however, they would be expected to influence similarly both heterojunctions with NiO and Cu_2_O, which is not the case. The ideality factor increases with decreasing Cu_2_O thickness and the concomitant decrease in the device shunt resistance, as can be seen in the dark *j-V* curves of Figure S4 for devices with 100 and 50 nm thick Cu_2_O. The main contribution to the large ideality factor is assumed to arise from the field-assisted recombination current at the Ga_2_O_3_/Cu_2_O interface due to the lowering of the potential barrier of traps or trap-assisted tunnelling at defect levels in the depletion region. With the increase in the forward bias, the electric field at the depletion region is reduced, decreasing these current contributions, which translates into an increased ideality factor [52,53]. 

Another important conclusion from the *j-V* characterization of the heterojunctions is that the ITO/Ga_2_O_3_ junction should have a low energy barrier for electrons to explain the observed transport characteristics. This is in contradiction to the measured large WF difference between ITO and Ga_2_O_3_ (0.6 eV), which should lead to a blocking of the electron transport and to very low currents. The question therefore arises regarding the reason for the observed unimpeded transport characteristics. An explanation is based on a significant amount of work in the literature on the type of contact between ITO and *β*-Ga_2_O_3_. Carey et al. reported ohmic contact between an *n*-type *β*-Ga_2_O_3_ wafer, with a carrier concentration of ~3 × 10^17^ cm^−3^, and Ti/Au, through an intermediate 10 nm thick, sputtered ITO layer. While Ga_2_O_3_/Ti/Au contacts remained of Schottky type after thermal rapid annealing at 600 °C, Ga_2_O_3_/ITO/Ti/Au contacts showed ohmic characteristics after annealing at 500 °C, dramatically improving the electron transport across the heterointerface [54]. The creation of an ohmic contact was attributed to the interdiffusion of In, Sn, and Ga at the heterojunction. Xia et al. [55] showed the reaction of sputtered ITO with highly doped Ga_2_O_3_ at temperatures > 300 °C. TEM and EDX characterization showed a roughening of the heterointerface, associated with the presence of a wide reaction zone, where the In and Sn from the ITO diffuse into the Ga_2_O_3_, with a corresponding modification of the electrical junction characteristics. The reaction zone significantly increased after annealing at 400 °C, with the interface losing its integrity at 500 °C. These results are aligned with the present work, showing an unimpeded electron transport at the ITO/Ga_2_O_3_ interface, suggesting a low potential barrier for electrons. An intermixed interface is highly probable in view of the high substrate temperature used for the spray pyrolysis of Ga_2_O_3_ on ITO (380 °C) applied for the deposition duration of ~15 min but also the extended cooling-down phase, with ~15 min needed for the sample to reach 250 °C. 

To gain more insight on the involved heterointerfaces, *C-V* measurements were realized for the NiO and Cu_2_O devices, using the parallel capacitance, $C_{P}$, and parallel conductance, $G_{P}$, equivalent circuit. The measured capacitance and conductance, $C_{M}$ and $G_{M}$, were corrected for the series resistance, $R_{S}$, using the approach described in [56,57]. The $R_{S}$ is calculated from the capacitance and conductance values at strong accumulation ($C_{M,\;acc}$, $G_{M,\;acc}$) using the equation:

$$R_{S}=\frac{G_{M,\;acc}}{G_{M,\;acc}^{2}+\omega^{2}C_{M,\;acc}^{2}}$$

The $C_{p}$ and $G_{p}$ in the three-element model are calculated by the following equations:

$$C_{P}=\frac{(G_{M}^{2}+\omega^{2}C_{M}^{2})\cdot C_{M}}{\alpha^{2}+\omega^{2}C_{M}^{2}}$$

$$G_{P}=\frac{(G_{M}^{2}+\omega^{2}C_{M}^{2})\cdot \alpha }{\alpha^{2}+\omega^{2}C_{M}^{2}}$$

$$\alpha =G_{M}-(G_{M}^{2}+\omega^{2}C_{M}^{2})\cdot R_{S}$$

**Figure 10:**
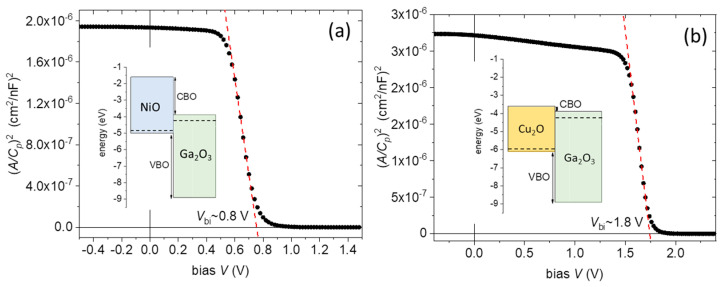
Mott–Schottky plots for (**a**) the Ga_2_O_3_(15)/NiO(20) junction and (**b**) the Ga_2_O_3_(15)/Cu_2_O(300) junction (red lines are linear fits), together with the corresponding band diagrams, showing the anticipated CBO and VBO in both cases.

Based on the above approach, the Mott–Schottky plots ${\left(\frac{A}{C_{P}}\right)}^{2}$ vs. V for the NiO and Cu_2_O junctions and for f = 10 kHz are shown in Figure 10a,b, respectively. The plots show a distinct linear region that corresponds to junction depletion. From the fit and extrapolation of the linear portion of the plots, the built-in potential, $V_{bi},$ can be extracted, which is ~0.8 V for the NiO junction and ~1.8 V for Cu_2_O. The $qV_{bi}$ corresponds to the difference in the Fermi levels between the *n* and *p* sides of the junction. The value of 0.8 eV is not far from the WF difference between Ga_2_O_3_ and NiO found from the Kelvin probe measurements, while the 1.8 eV is much larger than the WF difference between Ga_2_O_3_ and Cu_2_O from the Kelvin probe. However, as mentioned before, the formation of a CuO surface layer does not allow the estimation of the correct WF of Cu_2_O by the Kelvin probe. So, the value of ~6.0 eV extracted from the Mott–Schottky plot, considering the 4.2 eV as the WF of Ga_2_O_3_, is assumed to be a representative WF value for the Cu_2_O layer. 

## 4. Conclusions

In conclusion, it was shown that high-quality *n*-Ga_2_O_3_, *p*-NiO and *p*-Cu_2_O layers can be deposited by spray-pyrolysis (Ga_2_O_3_) at high temperature and RF (NiO) and reactive DC sputtering (Cu_2_O) without substrate heating, with properties that make them suitable as electron- and hole-transport layers, respectively, in different types of solar cells, such as perovskite and organic, as well as optoelectronic devices. Type II heterojunctions are formed between the Ga_2_O_3_ and NiO or Cu_2_O. Ga_2_O_3_/NiO junctions show large offsets for both conduction and valence bands, while, for Ga_2_O_3_/Cu_2_O, a large offset is only present for the valence band. The rectification is high for both types of junctions. Their transport characteristics can be described by a generation-recombination channel in the first case, with an ideality factor between 1 and 2, while, in the second case, high ideality factors above 3.5 suggest significant contributions from field-assisted recombination at increased trap density in the depletion region. Further, it was shown that a low resistance ITO/Ga_2_O_3_ contact is formed that does not hinder electron transport, despite the expectations from the band structure of the individual layers. This is assumed to be due to the interface intermixing during the high-temperature deposition of Ga_2_O_3_. Low resistance contacts to Ga_2_O_3_ are of interest for power electronic devices, apart from the applications aforementioned. Open circuit voltage values of ~220 and ~940 mV were achieved for the NiO- and Cu_2_O-based junctions, respectively. Ga_2_O_3_/NiO junctions absorb only in the UV region and can be applicable as photodiodes or transparent image sensors. Visible-active solar cells can be based on the Ga_2_O_3_/Cu_2_O heterojunction. At the current state, the short circuit current is too low for practical implementation but the optimization of the absorber thickness, as well as its structural and electronic properties (e.g., grain size and charge carrier mobility) can lead to significant improvements in performance. 

## Data Availability

Data are contained within the article and Supplementary Material.

## Supplementary Materials

The following supporting information can be downloaded at: https://www.mdpi.com/article/10.3390/nano14030300/s1, Figure S1: AFM images of the glass substrates; Figure S2: Initial screening of the effect of Ga_2_O_3_ thickness on the open circuit voltage, realized for samples with a Ga_2_O_3_ thickness gradient; Figure S3: *j-V* curve of heterojunction with inserted NiO layer between Cu_2_O and Au; Figure S4: *j-V* curves of heterojunctions with reduced Cu_2_O thickness.
