# Supplementary material for: Heterojunction Devices Fabricated from Sprayed n-Type Ga2O3, Combined with Sputtered p-Type NiO and Cu2O
Source: Nanomaterials (Basel). 2024 Feb 1;14(3):300. doi: 10.3390/nano14030300 (PMC10856998; doi:10.3390/nano14030300)
Supplement: Supplementary file 1 [file nanomaterials-14-00300-s001.zip › nanomaterials-2802740-supplementary.pdf]

SUPPLEMENTARY MATERIAL

Heterojunction devices fabricated from sprayed *n*-type Ga_2O_3, combined with sputtered *p*-type NiO and Cu_2O

Theodoros Dimopoulos,[1,*] Rachmat Adhi Wibowo,[1] Stefan Edinger,[1] Maximilian Wolf,[1] and Thomas Fix[2]

[1]Energy Conversion and Hydrogen Technologies, Center for Energy, AIT Austrian Institute of Technology, Giefinggasse 2, 1210 Vienna, Austria
[2]ICube Laboratory, Université de Strasbourg and Centre National de la Recherche Scientifique (CNRS), 23 rue du Loess, BP 20 CR, F-67037 Cedex 2 Strasbourg, France
*Corresponding author: theodoros.dimopoulos@ait.ac.at

1. AFM images of the glass substrates

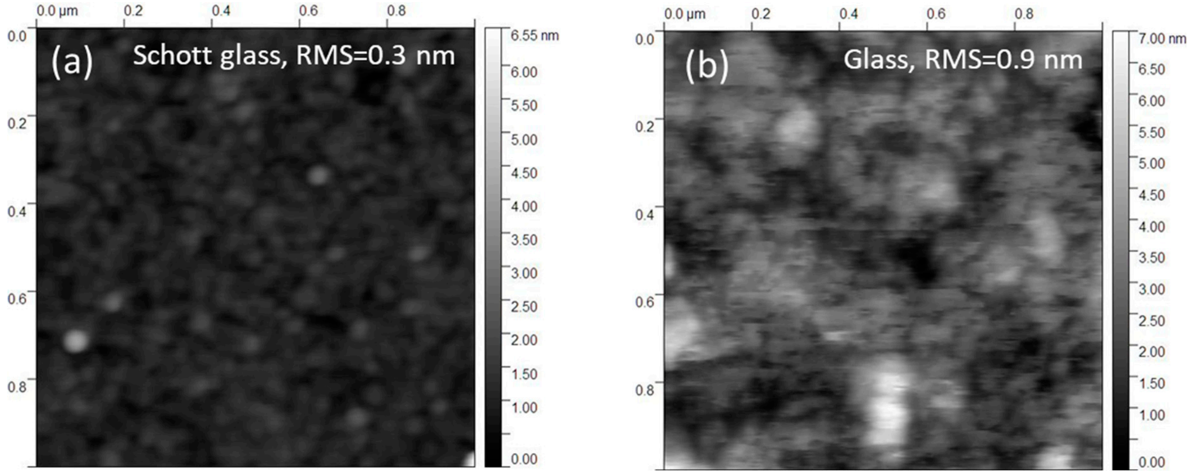

Figure S1. (a) AFM image of the bare borosilicate glass and (b) of the bare soda-lime glass.

2. Determination of the optimal Ga_2O_3 thickness for the heterojunctions

An initial screening phase showed that the optimal thickness of Ga_2O_3 is in the range of 15 nm. For this screening phase, we have used a thickness gradient of Ga_2O_3, from ~2 nm to ~37 nm, over a length of 7.5 cm, on ITO-coated glass. To achieve the gradient, the USP nozzle scan pattern had to be modified compared to the one for the samples with the uniform thickness, which can result to slightly different film properties, but the performance trends were assumed to hold. The gradient was coated with 200 nm of sputtered Cu_2O and Au contacts (dots of ~2 mm in diameter) were deposited through a shadow mask. The cells were measured under solar simulator with an automatized set-up. Figure S2 plots the best cell V_{oc} as a function of the thickness of Ga_2O_3. From this initial screening, the thickness of the Ga_2O_3 was selected at 15 nm, which corresponds to the maximum V_{oc}.

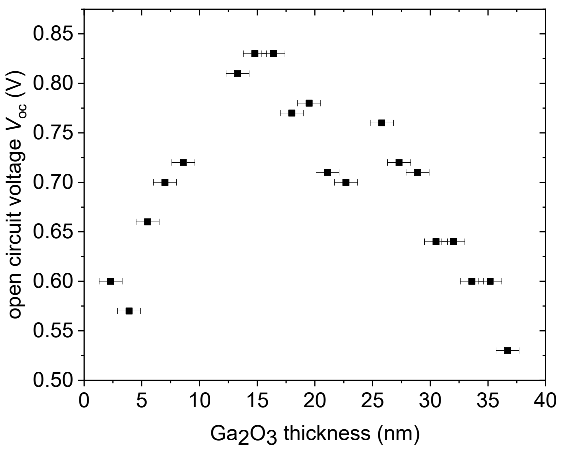

Figure S2. Initial screening of the effect of Ga_2O_3 thickness on the open circuit voltage, realized for samples with a Ga_2O_3 thickness gradient. From this screening, the thickness of Ga_2O_3 was selected at 15 nm.

3. J-V curve of heterojunction with inserted NiO layer between Cu_2O and Au

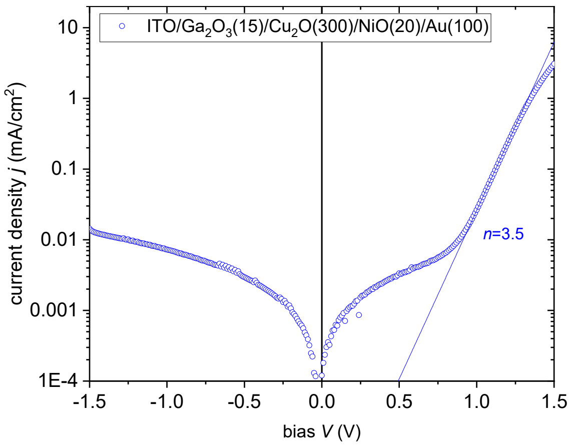

Figure S3. Dark j-V curve of heterojunction with inserted NiO layer between Cu_2O and Au, showing similar ideality factor to junctions without the NiO layer.

4. J-V curves of heterojunctions with reduced Cu_2O thickness

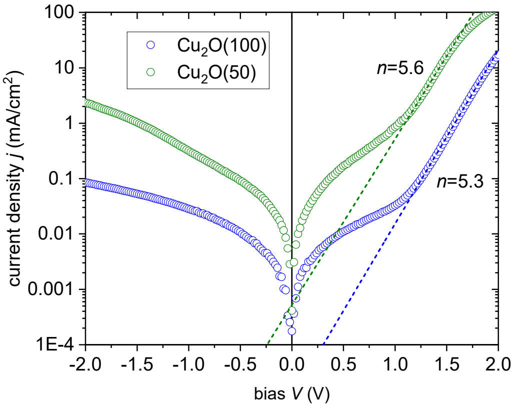

Figure S4. Dark *j-V* curves for heterojunctions with reduced Cu_2O(100) and Cu_2O(50) thickness, showing larger ideality factor and smaller parallel resistance.
